# Development of *B. carinata* with super-high erucic acid content through interspecific hybridization

**DOI:** 10.1007/s00122-021-03883-2

**Published:** 2021-07-16

**Authors:** Vicky Roslinsky, Kevin C. Falk, Roman Gaebelein, Annaliese S. Mason, Christina Eynck

**Affiliations:** 1 Saskatoon Research and Development Centre, Agriculture and Agri-Food Canada, Saskatoon, SK Canada; 2 Department of Plant Breeding, IFZ Research Centre for Biosystems, Land Use and Nutrition, Justus Liebig University Giessen, Giessen, Germany; 3 Department of Plant Breeding, INRES, University of Bonn, Bonn, Germany

## Abstract

**Key message:**

Disomic alien chromosome addition *Brassica carinata* lines with super-high erucic acid content were developed through interspecific hybridization with *B. juncea* and characterized using molecular, cytological and biochemical techniques.

**Abstract:**

*Brassica carinata* [A.] Braun (BBCC, 2*n* = 34) is a climate-resilient oilseed. Its seed oil is high in erucic acid (> 40%), rendering it well suited for the production of biofuel and other bio-based applications. To enhance the competitiveness of *B. carinata* with high erucic *B. napus* (HEAR), lines with super-high erucic acid content were developed through interspecific hybridization. To this end, a *fad2B* null allele from *Brassica juncea* (AABB, 2*n* = 36) was introgressed into *B. carinata*, resulting in a *B. carinata fad2*B mutant with erucic acid levels of over 50%. Subsequently, the *FAE* allele from *B. rapa* spp. yellow sarson (AA, 2*n* = 20) was transferred to the *fad2B B. carinata* line, yielding lines with erucic acid contents of up to 57.9%. Molecular analysis using the *Brassica* 90 K Illumina Infinium™ SNP genotyping array identified these lines as disomic alien chromosome addition lines, with two extra A08 chromosomes containing the *BrFAE* gene. The alien chromosomes from *B. rapa* were clearly distinguished by molecular cytogenetics in one of the addition lines. Analysis of microspore-derived offspring and hybrids from crosses with a CMS *B. carinata* line showed that the transfer rate of the A08 chromosome into male gametes was over 98%, resulting in almost completely stable transmission of an A08 chromosome copy into the progeny. The increase in erucic acid levels was accompanied by changes in the proportions of other fatty acids depending on the genetic changes that were introduced in the interspecific hybrids, providing valuable insights into erucic acid metabolism in *Brassica*.

**Supplementary Information:**

The online version contains supplementary material available at 10.1007/s00122-021-03883-2.

## Introduction

*Brassica carinata*, also known as Ethiopian or Abyssinian mustard, is a member of the *Brassicaceae* (mustard) family. It is an allotetraploid (genome BBCC, 2*n* = 34) formed through the interspecific hybridization between the diploid progenitor species *B. nigra* (BB, 2*n* = 16) and *B. oleracea* (2*n* = 18) (U 1935; Prakash et al. 2011). Current evidence suggests that it originated in the highland plateaus of Ethiopia and neighboring areas of East Africa and the Mediterranean coast (Gomez-Campo and Prakash 1999; Alemayehu and Becker 2002). While the cultivation of Ethiopian mustard as an oilseed and vegetable crop in East Africa dates back several millennia (Simmonds 1979), interest in this crop in other geographic regions is relatively recent. This interest primarily stems from the well-documented heat and drought tolerance of this crop (Cohen and Knowles 1983; Ferreres et al. 1984; Malik 1990), and its potential to serve as a dedicated feedstock crop for biofuel production and other industrial applications (Taylor et al. 2010, Marillia et al. 2014).

*B. carinata* seed oil is high in erucic acid, with proportions ranging from 31 to 46% in natural germplasm collections (Röbbelen and Thies 1980; Westphal and Marquard 1980; Becker et al. 1999; Warwick et al. 2006). Plant oils high in erucic acid are of interest for industrial purposes; erucic acid and its derivatives are important renewable raw materials for the oleochemical industry, with more than 1000 potential or patented applications (Scarth and Tang 2006). Erucic acid (*cis*-13-docosenoic acid, C22:1) is a straight-chained mono-unsaturated very-long-chain fatty acid (VLCFA) with 22 carbon atoms and a double bond at the *cis*-13 position of the carbon chain. Biosynthesis of this fatty acid involves chain elongation of oleic acid (*cis*-9-octadecenoic acid, C18:1) to gondoic acid (*cis*-11-eicosenoic acid, C20:1) and then to C22:1 (Sanyal et al. 2015). This reaction is catalyzed by ß-ketoacyl-CoA synthase (KCS), a membrane-bound enzyme that utilizes malonyl-CoA as the source of the two carbon atoms added in each cycle (Griffiths et al. 1998). KCS is encoded by the gene fatty acid elongase (*FAE*) (James et al. 1995; Lühs and Friedt 1997) and is the rate-limiting enzyme that plays a key role in determining the levels of erucic acid and other VLCFAs in seed oils (Millar and Kunst 1997). Early work in *B. carinata* proposed that C22:1 content is controlled by two loci in an additive manner with one allele present in each genome (Getinet et al. 1997). In agreement with this, it was demonstrated that two *FAE* genes determine the C22:1 content in the closely related species *B. napus* (Fourmann et al. 1998) and *B. juncea* (Venkateswari et al. 1999). Several studies, including experiments that involve interspecific hybrids, have demonstrated that the erucic acid content in Brassica seed oils is highly heritable (Getinet et al. 1997; Alemayehu and Becker 2001; Farhatullah et al. 2014).

Results from traditional breeding approaches in *B. carinata* suggest that C22:1 levels ranging from zero (Alonso et al. 1991; Getinet et al. 1994) to low (< 2%; Fernandez-Escobar et al. 1988; Velasco et al. 1995) to as high as 51% (Alemayehu and Becker 2001) are possible due to natural variation and through selection breeding, compared to <1 to 60% in non-canola types of *B. rapa* and *B. napus* (Lühs and Friedt 1994; Lühs et al. 1999) and <1 to 52% in *B. juncea* (Saikia et al. 2018). Several studies in various *Brassica* species have manipulated C22:1 levels via mutagenesis or transgenic approaches. Thus, chemical mutagenesis has been used to both reduce and elevate C22:1 levels in *B. carinata* (Barro et al. 2001). UV treatment has also been used to increase C22:1 levels from 42.8 to 49.5% (Barro et al. 2003). Both sense and antisense *FAE* constructs were used in *B. napus* (Zebarjadi et al. 2006) and *B. juncea* (Kanrar et al. 2006) to increase and decrease the C22:1 content, respectively. In *B. napus* cultivar CY2, an RNAi approach reduced the C22:1 content from 40 to 3%, with corresponding increases in C18:1 from 20 to 60% (Shi et al. 2015). C22:1 levels were increased in *B. carinata* by either a co-suppression or antisense construct of omega-6 fatty acid desaturase (*FAD2*) (Jadhav et al. 2005) through an increase in the amount of the substrate C18:1. Further, silencing of *FAD2* via a hairpin construct in combination with a *Crambe abyssinica FAE* construct in *B. carinata* resulted in levels of C22:1 as high as 56% (Mietkiewska et al. 2008).

While genetic transformation is a powerful means of effectively transferring genes across reproductive barriers (Cardi et al. 2017), genetically modified crops are facing great regulatory and trade challenges in contrast to crops developed through traditional breeding (Smyth 2016). Serendipitously, in the *Brassica* genus, several crop species have genomes in common and the ability to perform interspecific hybridizations allows for the transfer of useful adaptive traits between species for targeted crop improvement through conventional breeding (Katche et al. 2019). Interspecific crosses with the compatible allotetraploid species *B. napus* and *B. juncea* have resulted in *B. carinata* lines with decreased glucosinolate content (Márquez-Lema et al. 2008) and increased seed oil content (Sheikh et al. 2009), as well as increased overall genetic diversity (Sheikh et al. 2011). Similarly, C22:1 levels in *B. carinata* were significantly reduced through crosses to both *B. napus* (Fernandez-Escobar et al. 1988) and *B. juncea* (Getinet et al. 1994). On the other hand, *B. carinata* has been used as a donor for the transfer of a number of traits, such as blackleg (*Leptosphaeria maculans*) resistance to *B. napus* (Fredua-Agyeman et al. 2014; Navabi et al. 2010) and resistance to white rust (*Albugo candida*) and Alternaria blight (*Alternaria brassicae*) to *B. juncea* (Gupta et al. 2010). Several groups have utilized *B. carinata* to introduce yellow seed coat color into *B. napus* (Rashid et al. 1994; Meng et al. 1998; Rahman 2001). In addition, overall genetic diversity has been enhanced in both *B. napus* (Chen et al. 2010a, b) and *B. juncea* (Wei et al. 2016) through crosses with *B. carinata*. Interspecific crosses of *B. carinata* with diploid *Brassica* species have also been explored. The transfer of powdery mildew (*Erysiphe polygoni*) (Tonguç and Griffiths 2004) and black rot (*Xanthomonas campestris*) resistance (Sharma et al. 2017) from *B. carinata* to *B. oleracea* was examined in early backcross generations; both studies required embryo rescue. Crosses with *B. rapa* were found to be challenging and highly dependent on the accessions used (Jiang et al. 2007) and the direction of the cross (Choudhary et al. 2000), although successful crosses between *B. rapa* and *B. carinata* have been achieved in both directions (FitzJohn et al. 2007; Tian et al. 2010). In particular, successful crosses with diverse *B. rapa* genotypes including brown sarson, toria and yellow sarson have been achieved (Choudhary et al. 2000; Rahman 2001); yellow sarson is known to have erucic acid levels greater than 55% (Dorrell and Downey 1964).

Industrial applications demand an oil composition predominated by a single fatty acid. Compared to low-erucic *Brassica* oils, high erucic oils have a higher energy potential (Semenov et al. 2006) and are more desirable substrates for biodiesel (Vicente et al. 2005) and biofuel (Zhao et al. 2016) production. High erucic acid rapeseed (HEAR), currently the main feedstock for erucic acid used in industrial applications, contains about 50% of this fatty acid (Sanyal et al. 2015). For improved technical utility and enhanced marketability of carinata oil, it is desirable to increase the C22:1 content and to simultaneously decrease the content of C20:1, polyunsaturated fatty acids (PUFA, linoleic [C18:2] and linolenic [C18:3] acids) and saturated fatty acids (palmitic [C16:0] and stearic [C18:0] acids).

The purpose of the present study was to create a *B. carinata* breeding line with super-high C22:1 content through interspecific hybridization with *B. juncea* and *B. rapa*. Herein, we report the development of a *B. carinata* disomic A genome chromosome addition line, carrying the *fad2*B*/FAE* gene variants, with erucic acid levels of close to 60%, and its characterization using molecular, cytological and biochemical techniques. Further, we demonstrate the almost completely stable transfer of the chromosome addition through crosses with cytoplasmic male sterile *B. carinata* and the production of offspring via isolated microspore culture. This study provides the first account of the development of super-high erucic acid Ethiopian mustard through conventional breeding and contributes to the understanding of erucic acid metabolism in *Brassica.* It is also the first report to describe a *B. carinata-rapa* disomic chromosome addition line.

## Materials and methods

### Plant materials and breeding strategy

**Fig. 1:**
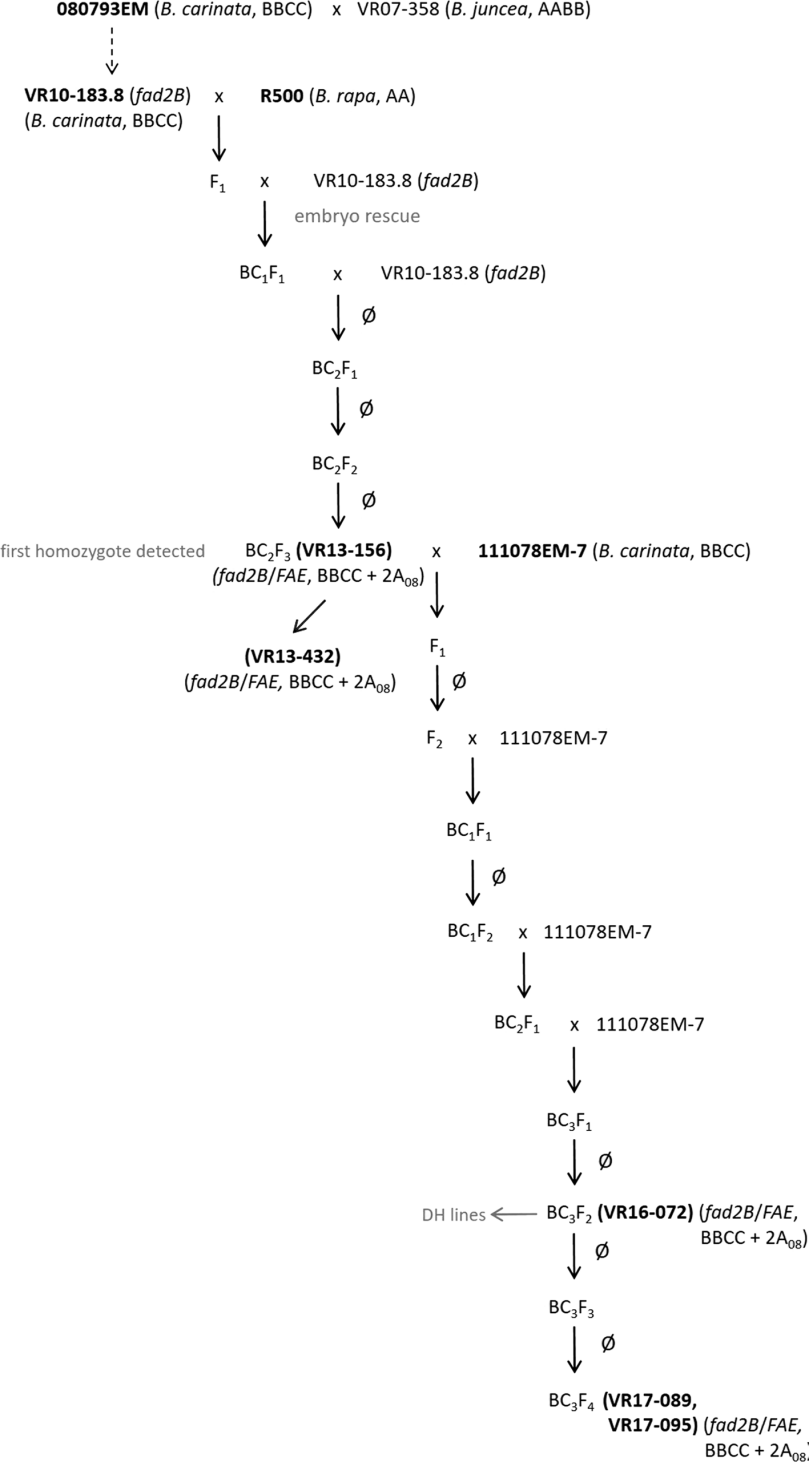
Schematic representation of the breeding strategy for the development of the super-high erucic acid *B. carinata* lines VR13-156 and VR17-095.* Note*: Line VR10-183.8 previously developed by (Roslinsky et al. 2011)

In previous work, the high erucic acid *B. carinata* breeding line 080793EM was crossed with canola-quality *B. juncea* line VR07-358 to increase the C18:1 content by introducing a *fad2B* null allele (Roslinsky et al. 2011). In the present study, the BC_3_F_3_ progeny line VR10-183.8 was crossed to the yellow sarson *B. rapa* line R500 in order to introduce the *B. rapa FAE* gene (*BrFAE*) (Fig. 1). The resulting F_1_ plants were backcrossed to VR10-183.8 and embryos rescued 16 days after pollination (DAP) according to the method described below. A second backcross was completed, followed by two rounds of self-pollination. The BC_2_F_3_ line VR13-156 was identified as a stable double mutant for the *fad2B* null allele and the *B. rapa*-derived *FAE* gene via molecular marker analysis, and was subsequently crossed to the doubled haploid (DH) *B. carinata* breeding line 111078EM-7 to improve total seed oil content and overall agronomics. Improvements to the *FAD2*-specific marker allowed for F_1_ lines to be used in the ensuing crossing procedure. Three backcrosses were carried out with 111078EM-7 as the recurrent parent, followed by three rounds of self-pollination. To determine the transfer rate of the *B. rapa FAE* gene during meiosis, BC_3_F_2_ line VR16-072 was used as the donor line for the production of microspore-derived plants. Additionally, individual BC_3_F_4_ lines were crossed to a cytoplasmic male sterile (CMS) line to examine pollen viability as well as segregation of the *BrFAE* allele in the F_1_ progeny. BC_3_F_4_ line VR17-095 was used for the molecular cytogenetic study. BC_3_F_4_ lines VR17-089 and VR17-095 were analyzed on a *Brassica* 90K Illumina Infinium™ SNP genotyping array. Parent material and progeny lines were grown in 2019 in the field in a randomized complete block design with four replicates alongside the two commercial *B. carinata* cultivars AAC A110 and AAC A120 (Agriculture and Agri-Food Canada—Saskatoon Research and Development Centre, Canada). Harvested seed was subjected to fatty acid analysis.

### Embryo rescue

F_1_ hybrids resulting from the cross between VR10-183.8 and yellow sarson *B. rapa* line R500 were used as the female in backcrosses with the recurrent parent VR10-183.8. BC_1_F_1_ plants were developed by embryo rescue followed by *in vitro* culture according to the protocol of Ayotte et al. (1987). Plantlets were transferred to soil and placed in a greenhouse.

### *FAE* and *FAD2* gene sequencing and marker development

**Fig. 2:**
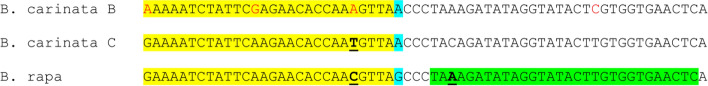
KASP primer positions in the *FAE* gene sequence. The targeted SNP at position 506 in the fragment is highlighted in blue, the sequence targeted by the selective primer in yellow and the sequence targeted by the common primer in green. The bases in red represent variation in the B allele, while bold and underlined bases were utilized for further selective amplification of the *BrFAE* sequence

Genomic DNA was isolated from young leaf tissue of the parents and progeny lines using a sodium dodecyl sulfate protocol as per Somers et al. (1998). The primer pair FAE F and FAE R (Online Resource 1) was designed to amplify all *FAE* alleles present in a sample, based on Gupta et al. (2004). All PCR reactions were carried out as follows: genomic DNA was used as the template for PCR amplification in a thermocycler with 35 cycles of 45 s for denaturation at 94 °C, primer annealing at 57 °C and 1 minute extension at 72 °C. The PCR products from the parental lines R500 and VR10-183.8 were subsequently cloned and sequenced; the expected bands were cloned into the pGEM-T Vector System I (Promega, Cat. No. A3600) and plasmids were extracted using the QIAprep Spin Miniprep Kit (Qiagen, Cat. No. 27106) following the manufacturer’s recommended protocols. All samples were sequenced at the National Research Council in Saskatoon, Canada. Screening of early generations for the presence of *BrFAE* was carried out with an *Hpa*I post-amplification digest using the above primer pair. The digest was run on a 2% agarose gel for 3 hours at 120 v to ensure adequate separation of fragments. To improve screening capacity, KASP primers (LGC Genomics) were developed for routine screening utilizing the *Hpa*I SNP position (Fig. 2) and implemented at the first backcross to recurrent parent 111078EM-7 and subsequent generations. The KASP reaction contains 100 ng genomic DNA, 4.0 μL KASP 2x Master Mix (LGC Genomics, Cat. No. KBS-1016-002-US) and 0.11 μL primer assay mix (Online Resource 1) in a total volume of 8 μL. All amplifications were performed in a CFX96 Real-Time Thermal Cycler (Bio-Rad Laboratories) using a touchdown PCR protocol as per the manufacturer’s recommended procedure.

Primer pair fad2B F and fad2B R (Online Resource 1) was designed to amplify the *FAD2B* allele. The amplification followed the same PCR protocol as described above, except that the annealing temperature was 64 °C. With this primer pair, selection against the amplified product was required to select the *fad2B* null allele. As more sequence information was obtained later in the pedigree, new primers were developed facilitating a co-dominant marker system. This new primer pair (fad2B F2 and fad2B R2) (Online Resource 1) amplified a region tightly linked to *FAD2* present in all samples. The PCR protocol is again similar to the one described above, with an annealing temperature of 55 °C. Amplified products for both primer pairs were separated on 2% agarose gels and visualized through ethidium bromide staining. All samples at the BC_2_F_1_ × 111078EM-7 stage and beyond were tested with the second *FAD2* primer set.

### Seed fatty acid analysis

Seed fatty acid composition of field-grown parental and progeny lines was analyzed according to Thies (1971) with the following modifications: methyl esters were separated by gas chromatography using an Agilent INNOWAX fused silica capillary column (7.5 m x 250 µm diameter x 0.5 µm film thickness) at 250 °C with hydrogen as the carrier gas. The methyl esters were prepared by base-catalyzed transesterification using sodium methoxide in methanol and hexane on oil extracted from 3.0 g of crushed seed.

### Genotyping

Parental and progeny lines were analyzed using the *Brassica* 90K Illumina Infinium™ SNP genotyping array as per the supplied protocol. Data analysis was performed utilizing the GenomeStudio software package v 2.0 (https://support.illumina.com/array/array_software/genomestudio/documentation.html) according to suggested best practices (Mason et al. 2017; Clarke et al. 2016).

### Isolated microspore culture

To determine the transfer rate of *BrFAE* during meiosis, BC_3_F_2_ line VR16-072, derived from cross VR13-156 × 111078EM-07, was used as the donor line for the production of microspore-derived plants. Isolated microspore culture was performed as described in Zatylny et al. (in press). Germinated embryos with true leaves and roots were transplanted to pots and transferred to the greenhouse.

### Molecular cytogenetics

Root tips were collected and chromosome spreads prepared from root tips following the protocol described in Gaebelein et al. (2019), adapted from Snowdon et al. (1997). Cells were observed using a Leica DRME microscope equipped with a Leica EL6000 fluorescent light source. Pictures were taken using a Leica DFC450 C camera and Leica Application Suite X software. Grayscale images showing the red and green fluorescent signals were processed to achieve optimal signal strength and contrast in ImageJ and combined. The combined image was then overlaid with the original grayscale image of the respective cell taken before hybridization using Photoshop CS6.

Labeled probe to detect B genome chromosomes was prepared using Roche Dig-Nick Translation Mix (Cat. No. 11745 816 910) according to the manufacturer’s specifications, using genomic *B. nigra* DNA as a template. Labeled probe to detect C genome chromosomes was prepared using the Invitrogen BioPrime DNA Labelling System (Cat. No. 18094011) according to the manufacturer’s specifications using DNA from BAC (Bacterial Artificial Chromosome) clone BoB014O06 (Howell et al. 2008) as template DNA, which contains the C genome-specific repetitive element *Bot1* (Alix et al. 2008), which is distributed across all C genome chromosomes. Fluorescent in situ hybridization was carried out according to the methods detailed in Gaebelein et al. (2019), as adapted from Leflon et al. (2016). 100 ng of B genome DNA and 200 ng of C genome-specific BAC DNA were used in the hybridization mix, and 2 ng/µl Anti-Digoxigenin-FITC (Roche, Cat. No. 11207741910) and 10 ng/µl Cy3-Streptavidin (GE-Healthcare, Cat. No. PA43001) dissolved in 5% BSA (Bovine Serum Albumin) were applied for visualization of the antibody-labeled DNA probes. DAPI (4′,6-diamidino-2-phenylindole)-containing medium (Vectashield H-1200) was used to visualize the chromosomes under UV-light excitation.

### Statistical analysis

Fatty acid profile data were analyzed by ANOVA using the Glimmix Procedure of SAS (SAS for Windows 9.3, SAS Institute, USA). Treatment mean comparisons were made using the Tukey–Kramer method at the *P* = 0.05 level.

## Results

### Development and characterization of super-high erucic acid *B. carinata*

In *B. carinata* BC_3_F_3_ breeding line VR10-183.8 a B1/A5 translocation event had removed a portion of chromosome B1 that contained the *FAD2B* allele (Roslinsky et al. 2011), as a result of the cross with canola-quality *B. juncea* (Potts et al. 1999). This translocation caused an increase in the C18:1 content, combined with an increase in C22:1, and reductions in both C18:2 and C18:3 levels. In the present study, crosses were initiated to combine the *fad2B* null allele with the *FAE* gene from the yellow sarson *B. rapa* line R500. The BC_1_ crosses were embryo rescued to obtain viable interspecific hybrids. All following generations naturally set varying amounts of seed. At the BC_2_F_3_ stage, the breeding line VR13-156 was identified to be a double mutant homozygous for both the *fad2B* null allele and the rapa-derived *FAE* allele. This line showed a further significant increase in C22:1 content compared to the parental line VR10-183.8. At the same time, this line displayed poor overall growth and seed set, and had a low total seed oil content. Three additional rounds of backcrossing to the elite *B. carinata* breeding line 111078EM-7 and three cycles of self-pollination were completed to develop true-breeding double mutant lines with improvements in agronomic and seed quality traits. BC_3_F_4_ line VR17-095 was selected based on superior seed yield in the greenhouse (unpublished data).

### *FAE* and *FAD2* sequence analysis and marker development

**Fig. 3:**
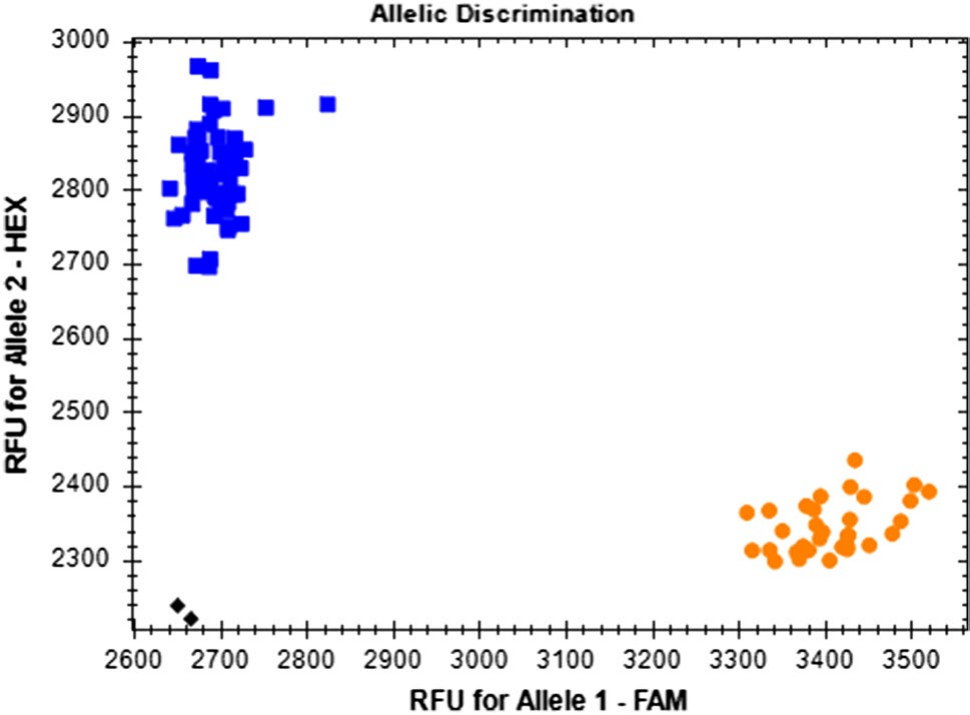
Bio-Rad CFX Maestro image of the FAE KASP marker. Blue squares represent samples homozygous for the A2 allele (no *BrFAE* allele), the orange circles represent both the homozygous A1 and heterozygous alleles (*BrFAE* allele present), and the black diamonds represent no template controls

A 1254 bp portion of the *FAE* genes from the breeding lines VR10-183.8 and R500 was amplified and sequenced using primers based on data from Gupta et al. (2004). A SNP was detected in an *Hpa*I digestion site at position 506 of the fragment which is present in both *B. carinata* endogenous *FAE* alleles, but absent in the R500 allele. Originally, this SNP was utilized in a post-amplification restriction digest that produced a unique 538 bp band specific for the *BrFAE* allele (Online Resource 2). To improve marker efficiency, this SNP was converted to a KASP marker. As three homoeologous copies of the *FAE* gene are present (*BcFAE-B*, *BcFAE-C* and *BrFAE*), both the selective and common primers were designed to preferentially amplify *BrFAE* to ensure adequate separation of the allele clusters and to capture all rare A genome insertion events (Fig. 2). This marker design is not typical of KASP markers and does not allow for the identification of heterozygous samples (Fig. 3).

**Fig. 4:**
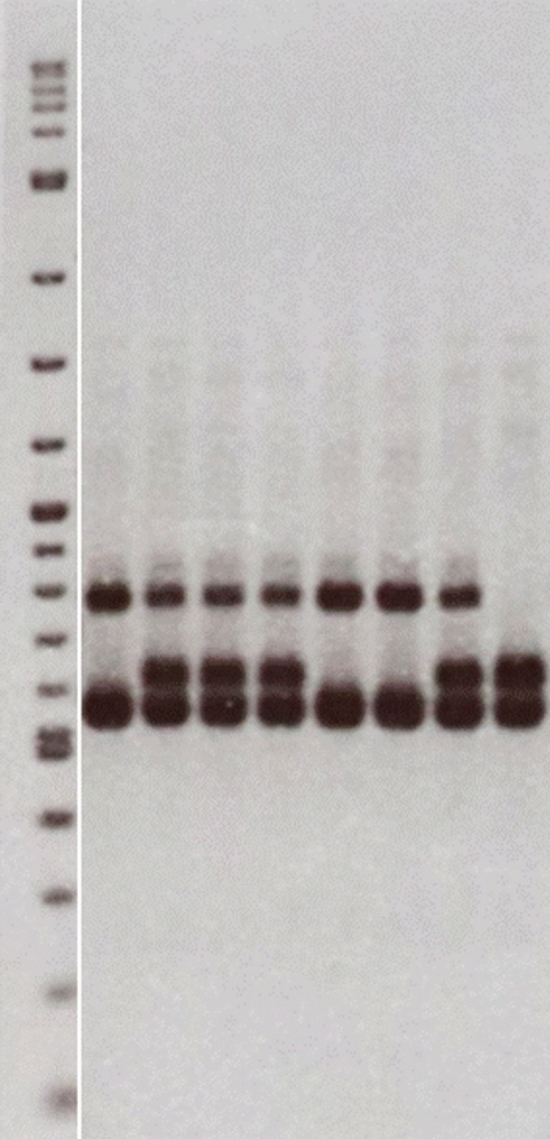
Gel image of samples analyzed with the co-dominant *FAD2B* marker. Lane 1 contains the 2 log DNA ladder (NEB), the bottom band is 100 bp, with 100 bp increments up to 1.0 kb; lanes 2, 6 and 7 represent samples homozygous for the *fad2B* null allele, lanes 3, 4, 5 and 8 represent samples heterozygous for the *fad2B* null allele, and lane 9 represents a sample with a wild-type *FAD2*B allele. The top band is associated with the *fad2B* null allele, the middle band with the carinata wild-type *FAD2*B allele and the bottom band with the *FAD2*C allele

The *fad2B* null allele was identified in canola-quality *B. juncea* and transferred to *B. carinata* (Roslinsky et al. 2011). The molecular marker (Genbank DQ777854) that was initially developed targets the *FAD2B* gene itself and amplifies a single 456 bp fragment (Online Resource 3). Because the desired genotype is the homozygous null allele and selection for the absence of amplification was required, selections could only be made in an F_2_ generation. This resulted in one additional round of selfing after each backcross. In order to save time and avoid the occurrence of false negatives, a second marker was designed based on the sequence of a non-coding fragment associated with the B1/A5 translocation (Figure S2). When analyzed with this marker, the *B. carinata* lines developed in this study contain a 473 bp fragment associated with the wild-type *FAD2B* allele, a 615 bp band associated with the A5 translocation and the *fad2B* null allele and a 440 bp fragment that appears to be associated with the *FAD2C* allele (Fig. 4). Because of the co-dominant nature of this marker, the selection of lines homozygous for the *fad2B* null allele is readily feasible.

The *FAE* and *FAD2* markers described herein were used throughout the pedigree to identify stable double mutants homozygous for the juncea-derived *fad2B* null allele and the rapa-derived *FAE* gene.

### Seed fatty acid analysis

The lines representing important steps in the pedigree alongside samples of two commercial *B. carinata* cultivars, AAC A110 and AAC A120, were grown in replicate in a field trial at Saskatoon, Saskatchewan, and their seed fatty acid profiles were analyzed by gas chromatography. The relative amount (percentage of total fatty acids) of the major fatty acids C18:1, C18:2, C18:3, C20:1, C22:1 and nervonic acid (C24:1) as well as the percentage of unsaturated fatty acids is presented in Table 1. The fatty acid profiles of cultivars AAC A110 and AAC A120 are representative of generic *B. carinata* germplasm, while the seed oil of breeding line 080793EM is characterized by elevated C22:1 levels (net increase of 4.9% and 3.9% compared to AAC A110 and AAC A120, respectively) and lower amounts of C18:1, C18:3 and C24:1. The results of the analysis show that *fad2B* line VR10-183.8 possesses a C18:1 content that is 3.3% higher than that in *B. carinata* parent line 080793EM. This increase in C18:1 is accompanied by a 4.3% increase in C22:1 and reductions in both C18:2 and C18:3. Both C20:1 and C24:1 levels remain unchanged. BC_2_F_3_ line VR13-156, the first line in the pedigree identified as homozygous for both the *fad2B* null allele and *BrFAE*, shows a further increase in C22:1 levels with values that are on average 11% above those in line 080793EM (57.9 vs. 46.9%) which corresponds to a proportional increase of 23%. Compared to VR10-183.8, line VR13-156 is characterized by an increased amount of C22:1 (57.9 vs. 51.2%) and proportional reductions of C18:3 and C20:1 levels, by 20.8 and 59.7%, respectively. Line VR13-432, a direct progeny line of VR13-156 derived through selfing, features a fatty acid profile very similar to that of its parent with a statistically significant difference, albeit small, only in C18:1 levels. Line 111078EM-7, used as the recurrent parent in backcrosses to VR13-156, to improve the seed oil content and agronomics of this line, has a fatty acid profile that is very similar to that of the two commercial cultivars included in this study. Interestingly, BC_3_F_4_ lines VR17-095 and VR17-89 have C22:1 levels that are 9.8% and 9.4% above those of the recurrent parent and 3.5 and 3.9% below those of donor parent VR13-156. In all *fad2B*/*FAE* lines the percentage of saturated fatty acids was slightly but significantly reduced when compared to that of the parent lines 080793EM and 111078EM-7, respectively.

**Table 1:** Mean values for fatty acids as percentage of total fatty acids of the seed oil of *Brassica carinata* parent and progeny lines grown in a field trial at Saskatoon, Saskatchewan, in 2019; *n* = 4 unless specified otherwise

Line	Fatty acids (% of total)													
	Total Sat.		C18:1		C18:2		C18:3		C20:1		C22:1		C24:1	
AAC A110^§§^	5.4	c	7.4	d	15.3	a	15.7	a	7.1	ab	42.0	f	2.9	ab
AAC A120^§§^	5.7	b	7.4	d	14.7	ab	14.6	b	7.7	a	43.0	ef	2.8	b
080793EM	5.9	a	6.3	f	15.4	a	13.0	c	6.2	b	46.9	d	1.9	c
VR10-183.8	5.2	c	9.6	a	10.8	e	12.0	d	6.7	b	51.2	c	1.8	cd
VR13-156^§^	4.9	d	9.0	b	11.7	cd	9.5	e	2.7	d	57.9	a	1.6	e
VR13-432	4.8	d	8.5	c	12.0	c	9.9	e	2.8	cd	57.4	a	1.7	de
111078EM-7	5.3	c	6.7	ef	14.2	b	15.6	a	6.2	b	44.6	e	3.0	a
VR17-095	4.8	d	6.8	ef	11.4	d	12.9	c	3.4	cd	54.4	b	2.7	b
VR17-089	4.8	d	6.9	de	11.9	cd	12.5	cd	3.5	c	54.0	b	2.7	b

Means within a column with different lowercase letters differ significantly at the *p* < 0.05 level

AAC A110, AAC A 120, 080793EM and 111078EM-7: generic *B. carinata* cultivars and breeding lines, respectively; VR10-183.8: *fad2B* null line derived from *B. carinata* 080793EM x *B. juncea*; VR13-156: double mutant line homozygous for *fad2B* and *B. rapa FAE*, BC_2_F_3_ derived from VR10-183.8 x R500; VR13-432: double mutant line homozygous for *fad2B* and *B. rapa FAE*, BC_2_F_4_ derived from selfing of VR13-156; VR17-089 and VR17-095: double mutant line homozygous for *fad2B* and *B. rapa FAE*, BC_3_F_4_ lines derived from VR13-156 x 111078EM-7

*Total sat.* Total saturated fatty acids include C12:0 (lauric), C14:0 (myristic), C16:0 (palmitic), C18:0 (stearic), C20:0 (arachidic), C22:0 (behenic) and C24:0 (lignoceric)

C18:1 = oleic acid, C18:2 = linoleic acid, C18:3 = linolenic acid, C20:1 = eicosenoic (gondoic) acid, C22:1 = erucic acid, C24:1 = nervonic acid

Fatty acids not listed include C16:1 (palmitoleic), C22:2 (docosadienoic), C22:3 (docosatrienoic), and trace amounts of other unidentified fatty acids

^§^ Three samples analyzed

^§§^ Two samples analyzed

### Genotyping

In order to determine the exact position and size of the *BrFAE* introgression, the BC_3_F_4_ lines VR17-089 and VR17-095 were analyzed using the *Brassica* 90K Illumina Infinium™ SNP genotyping array. *FAE* has previously been mapped to the A08 chromosome in *B. napus* (Fourmann et al. 1998; Delourme et al. 2006) and it was therefore expected that a portion of the A08 chromosome would be introgressed into the genome of *B. carinata*. The array data revealed that the entire *B. rapa* A08 chromosome was present, rendering VR17-089 and VR17-095 disomic alien chromosome addition lines (BBCC+2A_08_). The array data also showed that the distal portion of chromosome B1 was replaced with a distal portion of chromosome A5 (Figure S2). This re-arrangement led to the loss of the *FAD2B* allele, making VR17-089 and VR17-095 *fad2B/FAE B. carinata* disomic A-chromosome addition lines.

### Molecular cytogenetics

**Fig. 5:**
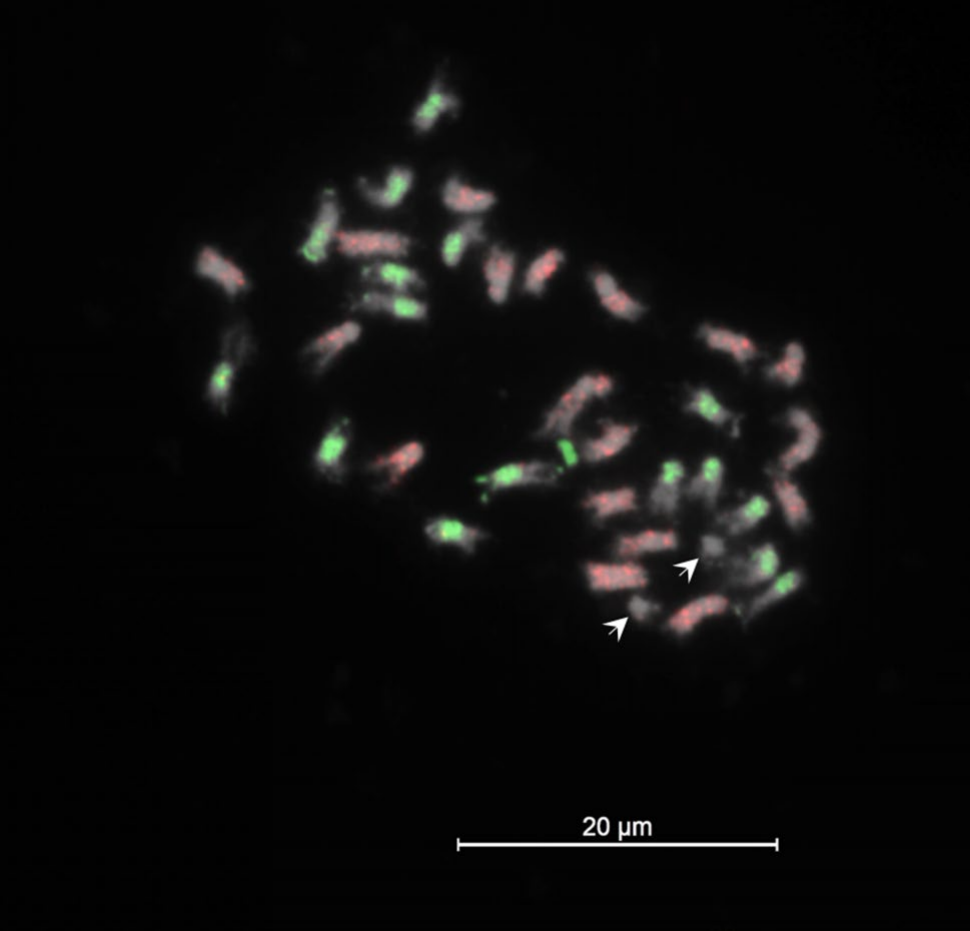
FISH/GISH stained mitotic cell of VR17-095 with a karyotype of 2*n* = 36. Green (fluorescein) signals represent chromosomes stained by probe derived from genomic DNA of *B. nigra*, identifying B genome chromosomes. Red signals (Cy3) represent chromosomes stained by probe derived from repetitive DNA sequence of *B. oleracea*, identifying C genome chromosomes. Two distinct small chromosomes (arrows) remain unstained, identifying them as putative A genome chromosomes

Unambiguous counts of 36 chromosomes were found in 50 cells from 23 independent plants of line VR17-095, supporting a stable karyotype of 2*n* = 36 in this line. Three microscope slides were successfully hybridized with probes specific to the *Brassica* B and C genomes, with sufficient signal strength, probe specificity and limited degradation of chromosomes on the slides. Fifteen chromosome spreads with good chromosome spreading and chromosome condensation were found to show 16 clear signals representing the B genome chromosomes, 18 clear signals representing the C genome chromosomes and two distinctly unstained chromosomes putatively representing the chromosome pair A08 introgressed from *B. rapa* (Fig. 5).

### Stability of the *B. rapa*-derived *FAE* introgression

To determine the transfer rate of the *BrFAE* gene during meiosis, BC_3_F_2_ line VR16-072 was used as a donor line for the production of microspore-derived plants. Of the 171 plantlets that were tested, 168 contained the *BrFAE* allele, which corresponds to a transfer rate of 98.25%. Further, in order to confirm that the pollen of the chromosome addition lines is viable and not selected against during pollination, crosses were carried out between 12 BC_3_F_4_ plants and a CMS *B. carinata* line. Seed set after pollination was normal. To confirm the transfer of the *BrFAE* allele, eight F_1_ plants from each of the 12 cross combinations were tested for its presence using the above described markers, with 95 of the 96 lines containing the *BrFAE* gene, equating to a transfer rate of 98.96%.

## Discussion

Erucic acid is one of several hundred unusual fatty acids produced by plants (Ohlrogge et al. 2018) and is found almost exclusively in triacylglycerols (TAGs) in the seed oil of the *Brassicaceae* family and the genus *Limnanthes* (meadowfoams) (Shi et al. 2015). C22:1 and its derivatives are important industrial feedstocks. Applications include the use of erucamide as a slip-promoting agent in plastic manufacturing and the production of high temperature lubricants, detergents, emulsifiers, photographic materials, as well as cosmetics and even pharmaceuticals (Leonard 1994; Sonntag 1995; Piazza and Foglia 2001; McVetty and Scarth 2002; Impallomeni et al. 2011). According to Li et al. (2012), it is estimated that a 10% increase in C22:1 content of total seed fatty acids results in a 50% reduction of the costs of erucamide production. The plethora of applications and the economics of production in combination with the need for improved technical utility and enhanced marketability of carinata oil have motivated our efforts in the development of *B. carinata* germplasm with super-high C22:1 levels. Here, we show that the level of C22:1 in *B. carinata* can be significantly increased through introgression of a *fad2B* null allele from *B. juncea* and introduction of an extra copy of *FAE* from *B. rapa*.

The enzyme FAD2 catalyzes the desaturation of C18:1 to C18:2, the first committed step in the synthesis of polyunsaturated fatty acids (Beisson et al. 2003). In line VR10-183.8, the loss of *FAD2B* causes a significant increase of the C18:1 proportion in seed TAGs compared to the seed oil of *B. carinata* parent line 080793EM, at the expense of the 18-carbon polyunsaturated fatty acids C18:2 and C18:3. Similarly, suppression of *FAD2* expression has been used successfully to increase the proportions of C18:1 in transgenic soybean and cotton as well as canola-quality *B. napus* and *B. juncea* lines (Stoutjesdijk et al. 2000; Kinney et al. 2002; Liu et al. 2002). It appears that the increased pool of C18:1 in VR10-183.8 was utilized by the endogenous fatty acid elongases (BcFAE-B, BcFAE-C), resulting in a simultaneous increase of the proportion of C22:1 to over 50% of total fatty acids. Thus, the reductive effect of the *fad2B* null allele on C18:1 desaturation may have enhanced substrate availability for the biosynthesis of C22:1 via elongation. This finding is in agreement with results from earlier work in *Arabidopsis thaliana* (Okuley 1994), *B. rapa* (Bao et al. 1998) and *B. napus* (Domergue et al. 1999). Jadhav et al. (2005) downregulated the expression of *FAD2* in *B. carinata* using co-suppression and antisense silencing approaches. While the resulting transgenic carinata lines had significantly increased C22:1 levels, none reached levels above 46% of total fatty acids. In addition, the effectiveness of these particular transgenic strategies is variable and unpredictable and they require the production of large transgenic populations to obtain an adequate number of lines showing sufficient levels of target gene suppression (Knutzon et al. 1992; Kinney 1996; Liu et al. 2002). Interestingly, in contrast to our results and the above cited studies, downregulation of *FAD2* in *Crambe abyssinica* by RNAi did not increase the C22:1 content, suggesting that oleate availability is not a rate-limiting factor in the biosynthesis of this fatty acid in crambe (Li et al. 2012; Cheng et al. 2013).

Line VR13-156, which in addition to the *fad2B* null allele possesses a heterologously expressed *B. rapa FAE* gene, exhibited an additional increase in C22:1 levels to just under 58%. Millar and Kunst (1997) were the first to show that introducing *A. thaliana FAE1* in *A. thaliana* or *Nicotiana tabacum* (tobacco) tissue void of significant quantities of VLCFAs resulted in their accumulation and that increasing gene dosage through introduction of extra copies of *FAE1* resulted in higher levels of these fatty acids. Since then, various *FAE* genes encoding enzymes with different acyl-CoA specificity have been identified, cloned and overexpressed in plants to increase C22:1 levels in the seed oil. Thus, overexpression of a *FAE* gene from *Tropaeolum majus* (garden nasturtium) in *A. thaliana* led to an increase in C22:1 proportions in the seed oil of up to eight-fold (Mietkiewska et al. 2004). In the present study, the very high proportion of C22:1 in the seed oil of *fad2B/FAE* line VR13-156 was associated with lower proportions of C18:3 and C20:1 compared to seed oil of VR10-183.8. This indicates that the new heterologous BrFAE acts in concert with the endogenous *B. carinata* FAE enzymes in elongating C18:1 to C22:1, which would limit the pool of C18:1 available for desaturation, as suggested by Mietkiewska et al. (2007). BrFAE may also be more efficient than the endogenous *B. carinata* FAE enzymes in elongating C18:1 to C22:1, a hypothesis corroborated by the naturally very high C22:1 proportions in seed oil of yellow sarson *B. rapa* line R500 (Dorrell and Downey 1964), the source of *BrFAE* in this study. Further, it is tempting to speculate that the BrFAE enzyme shows stronger elongase activity with gondoyl-CoA than with oleoyl-CoA, explaining the decrease in C20:1 levels in VR13-156 compared to those in VR10-183.8. The co-expression of elongases with different but complementary substrate preferences has been used successfully in a transgenic approach to increase the production of C22:1 in seeds of *B. carinata* (Mietkiewska et al. 2004), albeit not to the levels reported in this study.

While the introgression of the *fad2B* null allele alone results in a significant increase in the C18:1 content in VR10-183.8 compared to parent line 080793EM, the simultaneous introduction of this gene and *BrFAE* into wild-type 111078EM-7 did not yield significant changes in the content of this fatty acid in the lines VR17-089 and VR17-095. This may be explained by the immediate elongation of the increased pool of C18:1 through the heterologous BrFAE in seeds of the double mutant lines. This proposition is substantiated by the observation of Mietkiewska et al. (2007) that the C18:1 elongation pathway has a somewhat stronger ‘metabolic pull’ over C18:1 desaturation in developing seeds.

To the best of our knowledge, the current introgression of the *B. juncea fad2B* null allele and *FAE* gene from *B. rapa* has resulted in the highest level of C22:1 documented thus far for *B. carinata* seed oil. In comparison, the best result obtained through transgenic manipulations of *B. carinata* was observed in an RNAi *FAD2*/Crambe *FAE* transgenic line with 56% C22:1 (Mietkiewska et al. 2008). The C22:1 levels in double mutant lines VR17-089 and VR17-095 are lower than those in VR13-156 very likely because the recurrent parent 111078EM-7 they originated from also has a lower C22:1 content than line 080793EM which was used in the first cross with *B. juncea*. This stresses the importance of giving consideration to the level of the target fatty acid in the genetic background to be improved, in this case the recurrent parent, when attempting to develop germplasm with substantially increased proportions of that fatty acid.

Theoretically, the highest level of erucic acid that can be achieved in *B. carinata* seed oil through conventional breeding is 66.7%. This is because as in most *Brassicaceae*, in *B. carinata* the endogenous lysophosphatidic acid acyltransferase (LPAT), the enzyme responsible for placing acyl moieties in the sn-2 or middle position on the glycerol backbone during triacylglycerol biosynthesis via the Kennedy pathway (Kennedy 1961), cannot use erucic acid as a substrate (Frentzen 1993; Kuo and Gardner 2002; McVetty and Scarth 2002). In order to further increase the erucic acid content in our *fad2B*/*FAE* carinata lines, one option might be to introduce a diacylglycerol acyltransferase (DGAT) from a closely related *Brassica* species. DGATs catalyze the final step of triacylglycerol synthesis and it has been shown that specialized DGAT2s play a key role in channeling unusual acyl groups into TAGs (Shockey et al. 2006; Burgal et al. 2008; Li et al. 2010). In this scenario, the addition of an extra *Brassica* DGAT2 gene with high activity toward erucic acid would allow the plant to more efficiently channel erucic acid into TAG, as discussed by Demski et al. (2019).

In the present study, the introgression of *BrFAE* into the *B. carinata* genome was achieved through the transfer of the entire *B. rapa* A08 chromosome during the process of backcrossing. Almost completely stable *fad2B/FAE B. carinata* alien chromosome addition lines carrying two copies of chromosome A08 were produced and maintained. Alien chromosome addition lines in general are characterized by the addition of one (monosomic) or two (disomic) copies of a certain chromosome from one species to the genome of another species (Budahn et al. 2006). In *Brassica*, alien chromosome addition lines have been used extensively to assign linkage groups to chromosomes, study genomic relationships within the genus and to transfer agronomically important traits between species (Budahn et al. 2006; Chen et al. 1997; Fan and Tai 1985; McGrath and Quiros 1990; Quiros et al. 1987; Struss et al. 1991), with numerous studies particularly on the development and characterization of *B. napus*-*nigra* monosomic chromosome addition lines (AACC + 1B) (Chevre et al. 1997; Jahier et al. 1989; McGrath and Quiros 1990; Struss et al. 1991, 1992; Zhu et al. 1993). Successful examples of gene introgression from more distantly related species through monosomic addition lines include the transfer of resistance to *Leptosphaeria maculans* from *Sinapis arvensis* (wild mustard) (Snowdon et al. 2000) and resistance to the beet cyst nematode from *Raphanus sativus* (oil radish) (Peterka et al. 2004) into *B. napus*. While monosomic addition lines have proven to be useful in the dissection of genomes and studying chromosomal homoeologies, their practical utility in plant breeding programs is limited by segregation of the alien chromosome in offspring and potential loss of chromosome integrity by homoeologous recombination (Budahn et al. 2006). Thus, within the *Brassica* genus, transmission rates of alien monosomic chromosomes vary greatly and are usually below 50% (Budahn et al. 2006; Chen et al. 2010a, b; Chevre et al. 1997).

In contrast, disomic addition lines, which have been reported less frequently, display higher stability and fertility (Chen et al. 2010a, b; Chevre et al. 1991; Quiros et al. 1987; Wang et al. 2006; Zhang et al. 2013). To the best of our knowledge, our report is the first to describe a *B. carinata-rapa* disomic chromosome addition line. The transfer of the *B. rapa* A08 chromosome into the carinata genome is clearly demonstrated by the results of the *Brassica* 90K Illumina Infinium™ SNP genotyping array and further supported by the molecular cytogenetic analysis. Thus, considering the relatively unambiguous chromosome counts based on multiple plants, a stable karyotype of 36 chromosomes can be assumed for the interspecific hybrids, as shown for line VR17-095. The results of the molecular cytogenetic analysis suggest that two of the 36 chromosomes are indeed A genome chromosomes, for a karyotype of 2*n* = 34 + 2A.

The transfer rate of the *BrFAE* gene can be used to directly extrapolate the transmission rate of chromosome A08 as *FAE* is located on this chromosome (Fourmann et al. 1998; Delourme et al. 2006). It is well known that chromosome transmission rates are generally higher through female gametes than via male gametes (Budahn et al. 2006; Multani et al. 1994; Singh et al. 1998; Zhang et al. 2013); therefore, the focus of this study was on determining the transmission rate of chromosome A08 for male gametes by producing microspore-derived progeny and hybrid offspring by conducting crosses with a CMS *B. carinata* line. Both experimental approaches yielded rates of transmission of over 98%, suggesting virtually stable addition of A08 to the *B. carinata* genome. In comparison, *B. napus*-*B. nigra* disomic chromosome addition lines showed transmission rates for different nigra chromosomes between 50 and 100% (Chevre et al. 1991), indicating that the degree of transmission can vary depending on the species and chromosome under investigation.

A search on Ensembl Plants (https://plants.ensembl.org/index.html) revealed that chromosome A08 from *B. rapa* harbors additional genes that play a role in lipid metabolism, such as desaturases *FAD4* and *FAD6* as well as an acyltransferase and *LPAT5* (lysophosphatidic acid acyltransferase 5). However, it appears that these genes do not have a major impact on the fatty acid profile in the *fad2B/FAE B. carinata* disomic A-chromosome addition lines as the seed oil composition phenotype of these lines is consistent with that of *FAE1* over expression lines in other plants (Kunst 1997; Mietkiewska et al. 2004). It is also worth noting that *FAD2* and *FAD3*, the major genes involved in the synthesis of polyunsaturated fatty acids (Beisson et al. 2003), are located on chromosomes A03, A04 and A05, and not on A08.

In conclusion, this study has shown that the development of super-high erucic acid Ethiopian mustard is possible through traditional interspecific hybridization. To our knowledge, the results presented herein currently represent the highest accumulation of erucic acid achieved in *B. carinata* seeds. Although the lines we developed are not yet suitable for introgression into elite *B. carinata* breeding lines for cultivar development, because of the lack of completely stable introgression of the A08 chromosome, our results nevertheless make *B. carinata* an increasingly attractive alternative to HEA *B. napus* as a feedstock crop for industrial applications. In order to achieve a 100% stability of the *FAE* introgression, an attempt to introgress the *FAE* gene into the C genome through non-homologous recombination between A08 and a *B. carinata* C chromosome using intraspecific hybridization between the *fad2B/FAE B. carinata* disomic A-chromosome addition lines and resynthesized *B. carinata* is ongoing. Utilizing available *B. rapa* whole genome sequence information, a collection of A08-specific markers can be developed and, in conjunction with the existing *FAE* and *fad2B* markers, will allow for the selection of promising lines while reducing linkage drag.

## Electronic supplementary material

Below is the link to the electronic supplementary material.

Supplementary file1 (PDF 329 kb)

Supplementary file2 (DOCX 13 kb)

Supplementary file3 (TIF 87 kb)

Supplementary file4 (XLSX 134 kb)

## Data Availability

All datasets generated for this study are included in the article/supplementary material.
