# Supplementary material for: Development of B. carinata with super-high erucic acid content through interspecific hybridization
Source: Theor Appl Genet. 2021 Jul 16;134(10):3167–81. doi: 10.1007/s00122-021-03883-2 (PMC8440251; doi:10.1007/s00122-021-03883-2)
Supplement: Supplementary file 1 — Supplementary file1 (PDF 329 kb) [file 122_2021_3883_MOESM1_ESM.pdf]

Online Resource 1: Primer pairs used in this study to amplify fatty acid-related genes in *Brassica*

Primer name	Sequence
FAE F	CATTACGTCATAACCAACC
FAE R	GCTAGATGAAGTGTTTCCAG
FAE KASP A1	GAAGGTGACCAAGTTCATGCTGAAAATCTATTCA AGAACACCAACGTTAG
FAE KASP A2	GAAGGTCGGAGTCAACGGATTGAAAATCTATTCA AGAACACCAATGTTAA
FAE KASP C	GAGTTCACCACAAGTATACCTATATCTTTA
fad2B F	CTCGGGCAGACCTTACCCCGAG
fad2B R	GTCCCATCGAACTGGTAATAGTC
fad2B F2	GCTTCTCGTAAGCATCACGAG
fad2b R2	AGGTTTCGAAGATTCAACTTC
