# Supplementary material for: Development of B. carinata with super-high erucic acid content through interspecific hybridization
Source: Theor Appl Genet. 2021 Jul 16;134(10):3167–81. doi: 10.1007/s00122-021-03883-2 (PMC8440251; doi:10.1007/s00122-021-03883-2)
Supplement: Supplementary file 2 — Supplementary file2 (DOCX 13 kb) [file 122_2021_3883_MOESM2_ESM.docx]

Online Resource 2: Predicted band sizes from the FAE marker post-amplification digest. Unique 538 bp band specific for the BrFAE allele from Brassica rapa is shown in bold.

| allele | Predicted band sizes (bp) | | | | | | |
| --- | --- | --- | --- | --- | --- | --- | --- |
| *BrFAE* | 44 |  |  |  | **538** | 672 |  |
| *BcFAE-B* | 44 |  | 460 |  |  |  | 750 |
| *BcFAE-C* |  | 78 |  | 504 |  | 672 |  |
