# Supplementary figures and images for: Development of B. carinata with super-high erucic acid content through interspecific hybridization
Source: Theor Appl Genet. 2021 Jul 16;134(10):3167–81. doi: 10.1007/s00122-021-03883-2 (PMC8440251; doi:10.1007/s00122-021-03883-2)

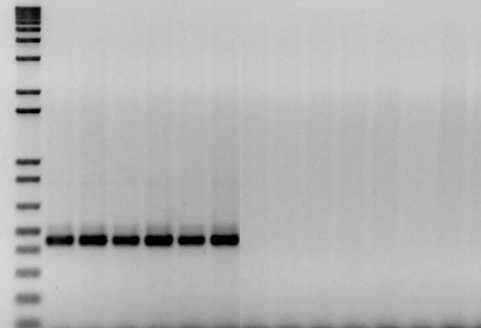

Supplement: Supplementary file 3 — Supplementary file3 (TIF 87 kb) [file 122_2021_3883_MOESM3_ESM.tif]
